# Supplementary material for: A population of Vasa2 and Piwi1 expressing cells generates germ cells and neurons in a sea anemone
Source: Nat Commun. 2024 Oct 10;15:8765. doi: 10.1038/s41467-024-52806-4 (PMC11464780; doi:10.1038/s41467-024-52806-4)
Supplement: Supplementary file 1 — Supplementary Information [file 41467_2024_52806_MOESM1_ESM.pdf]

A population of Vasa2 and Piwi1 expressing cells generates germ cells and neurons in a sea anemone

Supplementary Information

contains 10 Supplementary Figures
and Supplementary References

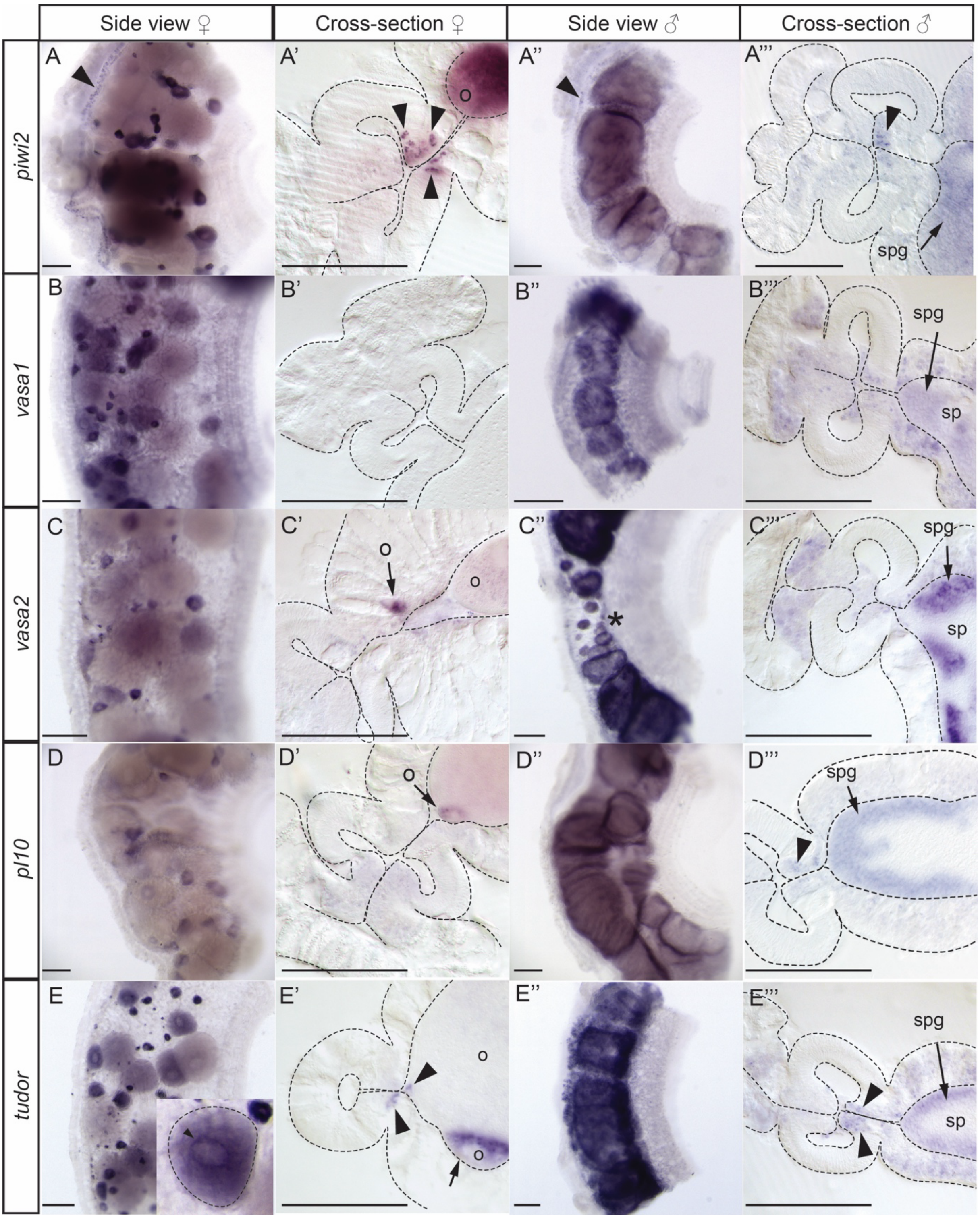

Supplementary Fig. 1. Expression of GMP marker gene orthologs in the adult gonadal mesentery of *Nematostella*. (A-E''') Whole mount tissue pieces and cross sections of female and male gonadal mesentery regions stained by ISH for *piwi2* (A-A'''), *vasa1* (B-B'''), *vasa2* (C-C'''), *pl10* (D-D''') and *tudor* (E-E''') genes. Expression is found in oocytes of different sizes ('o') and in spermatogonia ('spg'). In males, certain

regions of the gonad present smaller spermaries (C’’; asterisk). Expression of *piwi2* and *tudor* is also detected in basiepithelial cells that concentrate along the reticulate tract of the mesentery (A-A’’’, E’, E''; arrowheads). See also Supplementary Data 1 for a summary of expression regions. All scale bars correspond to 100µm. o: oocyte; sp: sperm; spg: spermatogonia. Experiments performed three times with similar results.

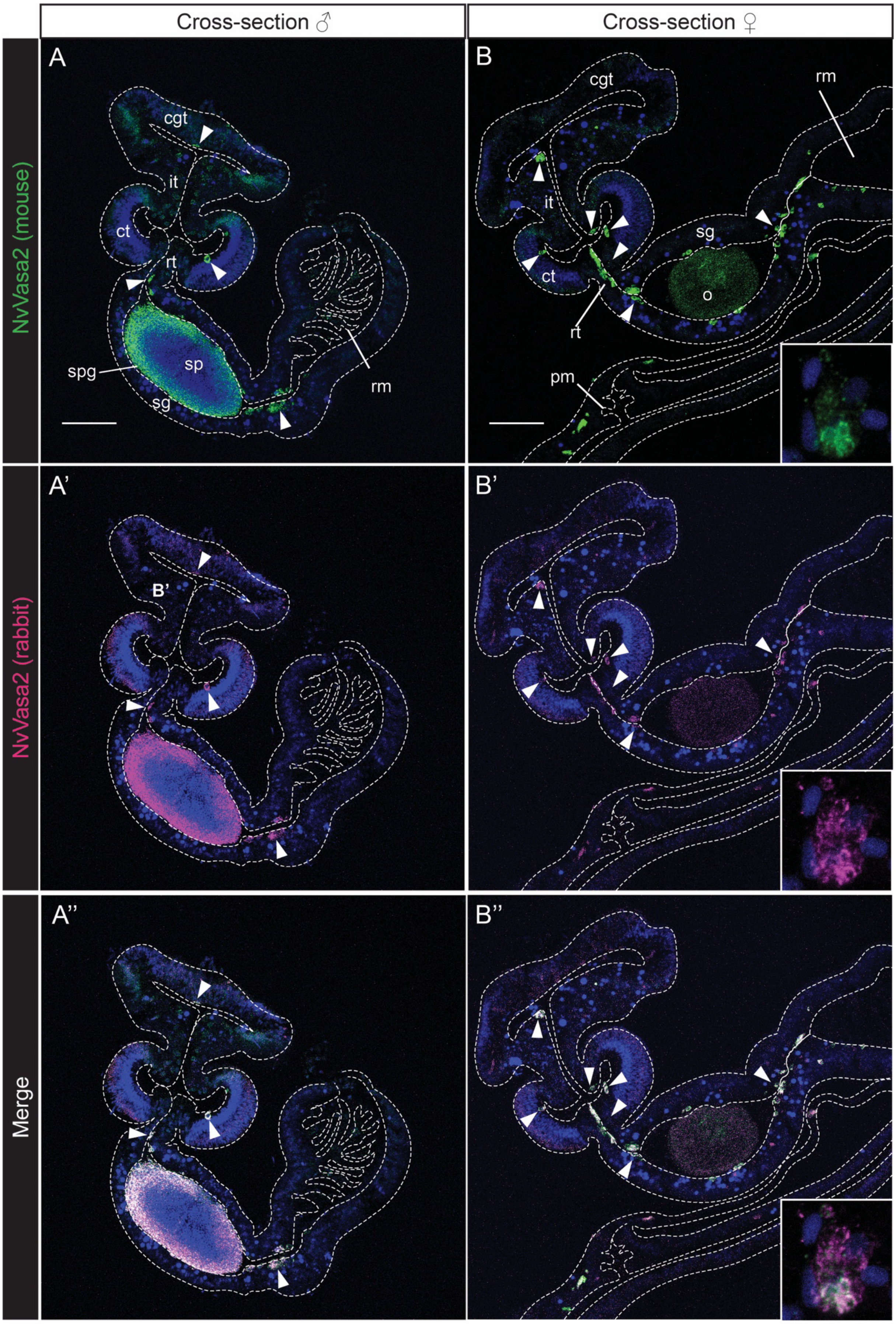

Cross-section ♂
Cross-section ♀
NvVasa2 (mouse)
NvVasa2 (rabbit)
Merge
A
cgt
it
ct
rt
spg
sp
sg
rm
B
cgt
rm
it
sg
ct
o
rt
pm
A'
B'
B'
A''
B''

Supplementary Fig. 2. Colocalization of two independently generated antibodies raised against *Nematostella* Vasa2. (A-B'') Confocal imaging stacks of cross sectioned young adult male (A-A'') and female (B-B'') gonadal mesentery regions immunostained for *Nematostella* Vasa2 with previously published monoclonal mouse anti-NvVasa2[1] (A-B; green) and polyclonal rabbit anti-NvVasa2[2] (A'-B'; magenta). The signal of both antibodies fully overlaps in developing spermatogonia (spg), oocytes (o) and in basiepithelial cells (arrowheads) located in the septal filament, gonad tract, and in the transition zone between gonad and retractor muscle (rm) (A-B'', arrowheads). Both antibodies label vesicular structures in specific cells near the parietal muscle region (inlets of B-B''). These cells are reminiscent of mucus cells previously reported in that region[3] and therefore likely the result of unspecific antibody binding. Notably, none of the [*vasa2::*mOr2]$_{low}$, [mOr2-Piwi1]$_{low}$ or [GFP(-P2A-Piwi1)]$_{low}$ cells in that region show any similar vesicular structures. Blue: Hoechst DNA dye. cgt: cnidoglandular tract; ct: ciliated tract; it: intermediate tract; o: oocyte; pm: parietal muscle; rm: retractor muscle; rt: reticulate tract; sg: somatic gonad; sp: differentiating sperm cells; spg: spermatogonia. Scale bars: 50µm. Experiment performed once.

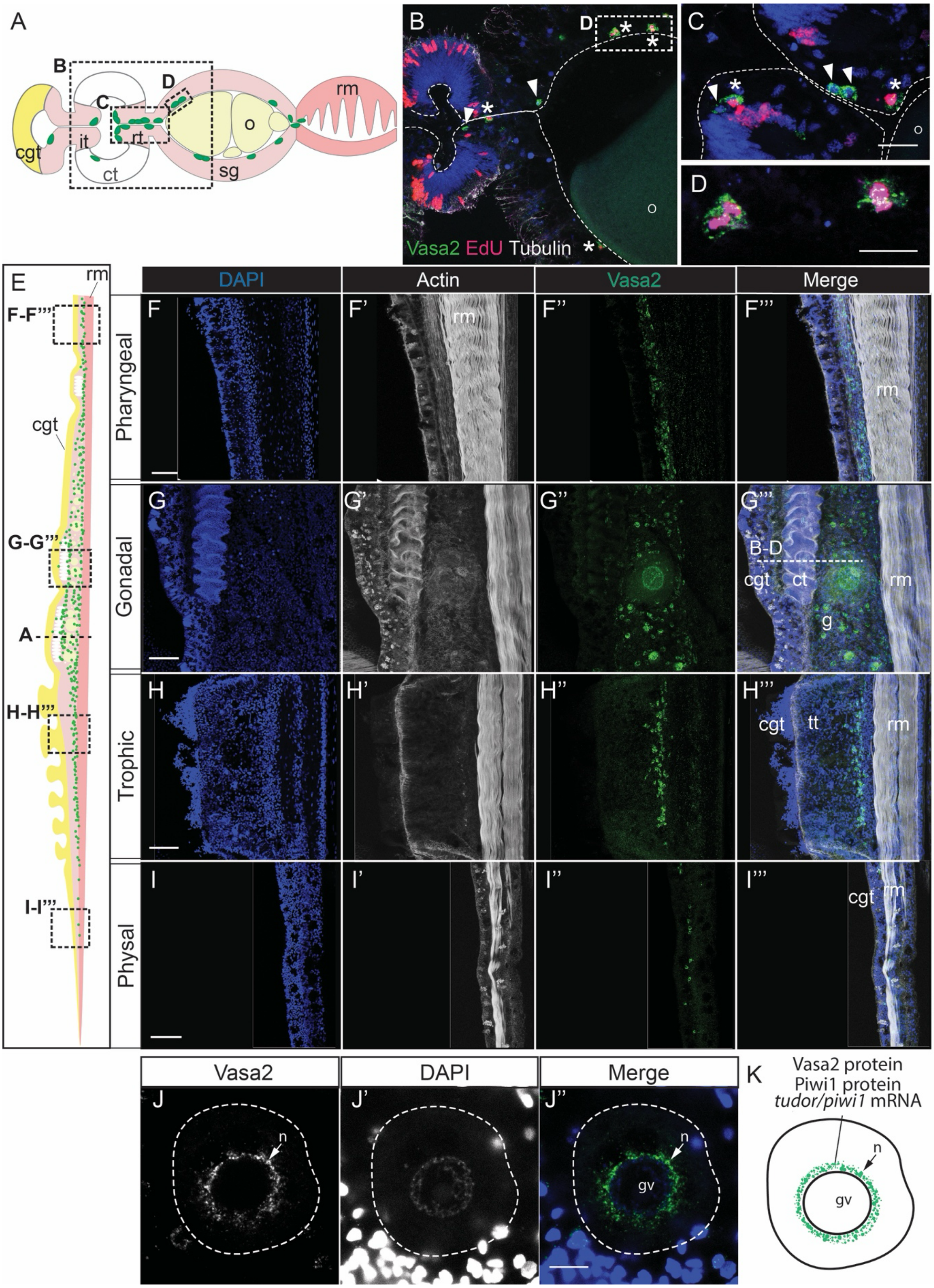

Supplementary Fig. 3. Dividing, basiepithelial Vasa2+ cells populate the mesenteries along the oral-aboral axis. (A, E) Schematics highlighting distribution of Vasa2+ cells (green dots) in a cross-section of the female gonadal region (A) or in

the lateral view of an entire adult female mesentery (E). Vasa2+ cells locate between the cnidoglandular tract (yellow, ‘cgt’) and the retractor muscle (dark pink, ‘rm’). Orientations: Oral to top and distal to left. **(B-D)** Confocal imaging stacks of cross-sectioned female gonadal mesentery immunostained for Vasa2 (green), α-Tubulin (white), and EdU-labelled for S-phase (3 days pulse, red). Small, basiepithelial Vasa2+ cells are found in the reticulate and gonad tracts (B, C; arrowheads & asterisks), partially displaying EdU incorporation (B, C; asterisks). Some Vasa2+/EdU+ cells show as duplets (D), suggesting they recently divided. **(F-J’’)** Confocal imaging stacks of pharyngeal (F-F’’’), gonadal (G-G’’’, J-J''), trophic (H-H’’’), physal (I-I’’’) regions of a whole adult female mesentery immunostained for F-actin (white) and Vasa2 (green). Note the dense concentration of F-actin fibers along the retractor muscle (‘rm’). Vasa2+ cells are less abundant towards the oral and aboral ends of the mesentery, with only few Vasa2+ cells present in the physal region (I’’). **(J-K)** Vasa2 protein (J, J'') colocalises with Piwi1 protein (see Fig. 1K), *piwi1* (see Fig. 1F) and tudor mRNA (see Supplementary Fig. 1E) to perinuclear granules (nuage; 'n') of developing oocytes as summarized schematically (K). Blue: DAPI DNA dye. cgt: cnidoglandular tract; ct: ciliated tract; g: gonad; n: nuage; o: oocyte; rm: retractor muscle, rt: reticulate tract; sg: somatic gonad. Scale bars: 10µm (C, D) and 50µm (F-I, J''). All experiments performed twice with similar results.

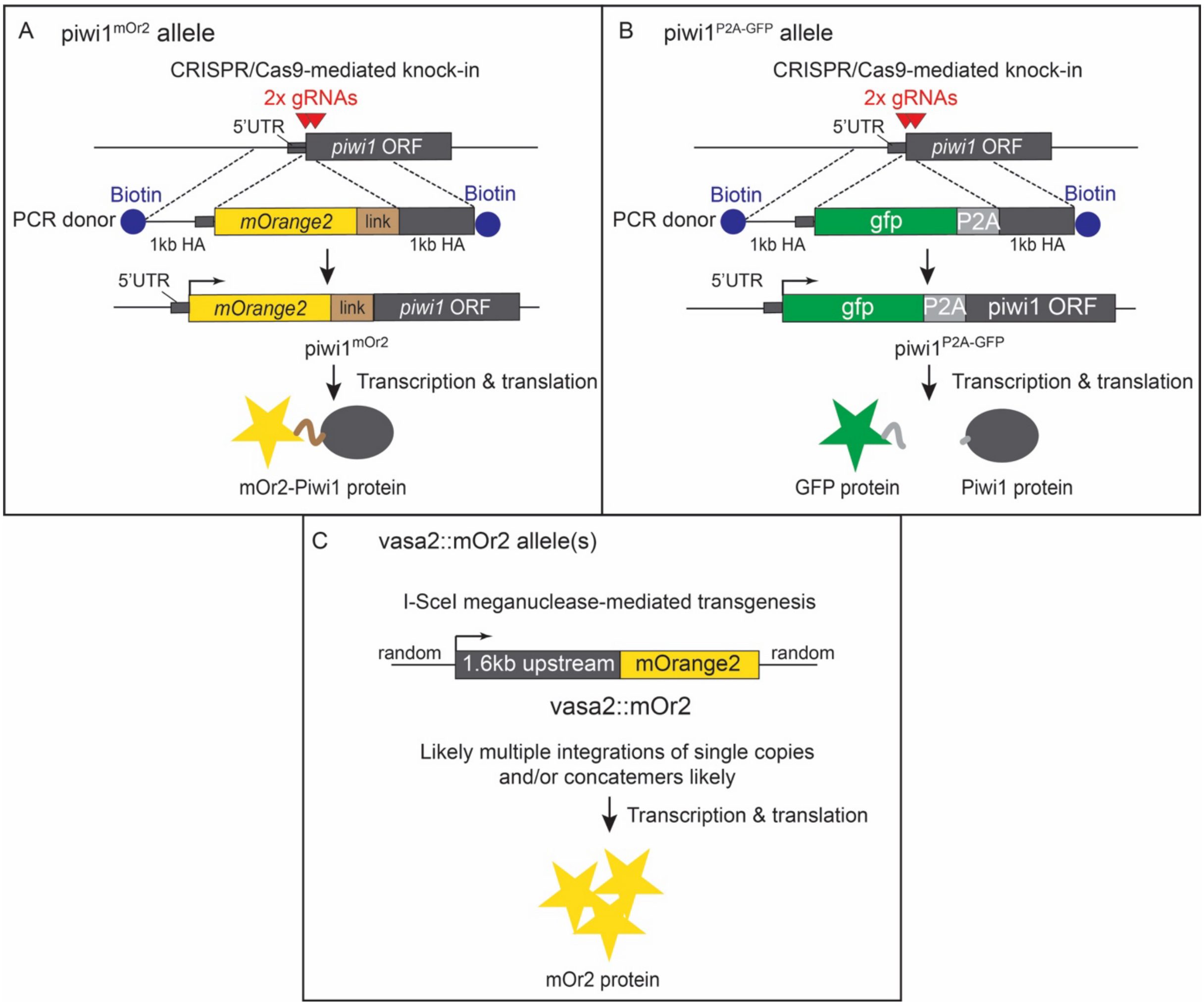

Supplementary Fig. 4. Schematized strategy for generating *piwi1^{mOr2}* (A), *piwi1$^{P2A\text{-}GFP}$* (B) and *vasa2::mOr2* transgenic lines. (A, B) *piwi1^{mOr2}* and *piwi1$^{P2A\text{-}GFP}$* alleles were generated by genomic knock-in using CRISPR/Cas9-mediated homologous recombination of biotinylated, double-stranded DNA donor constructs at the 5'-end of the *piwi1* open reading frame (ORF). Donor DNA was encoding for GFP or mOrange2 fluorophore and carried covalently bound biotin molecules at their 5'-end to protect against concatemerization. P2A is a viral peptide leading to ribosomal skipping and the translation of two separate polypeptides from a single mRNA. **(C)** The *vasa2::mOr2* transgenic lines was generated using I-SceI meganuclease-mediated, random integration of a construct driving mOr2 from a 1.6kb fragment that includes the putative promotor and cis-regulatory sequences of the endogenous *vasa2* gene. gfp: green fluorescent protein; gRNAs: guide RNAs, HA: homology arm, kb: kilobase, link: linker sequence ($GGGGS^2$); ORF: open reading frame; UTR: untranslated region.

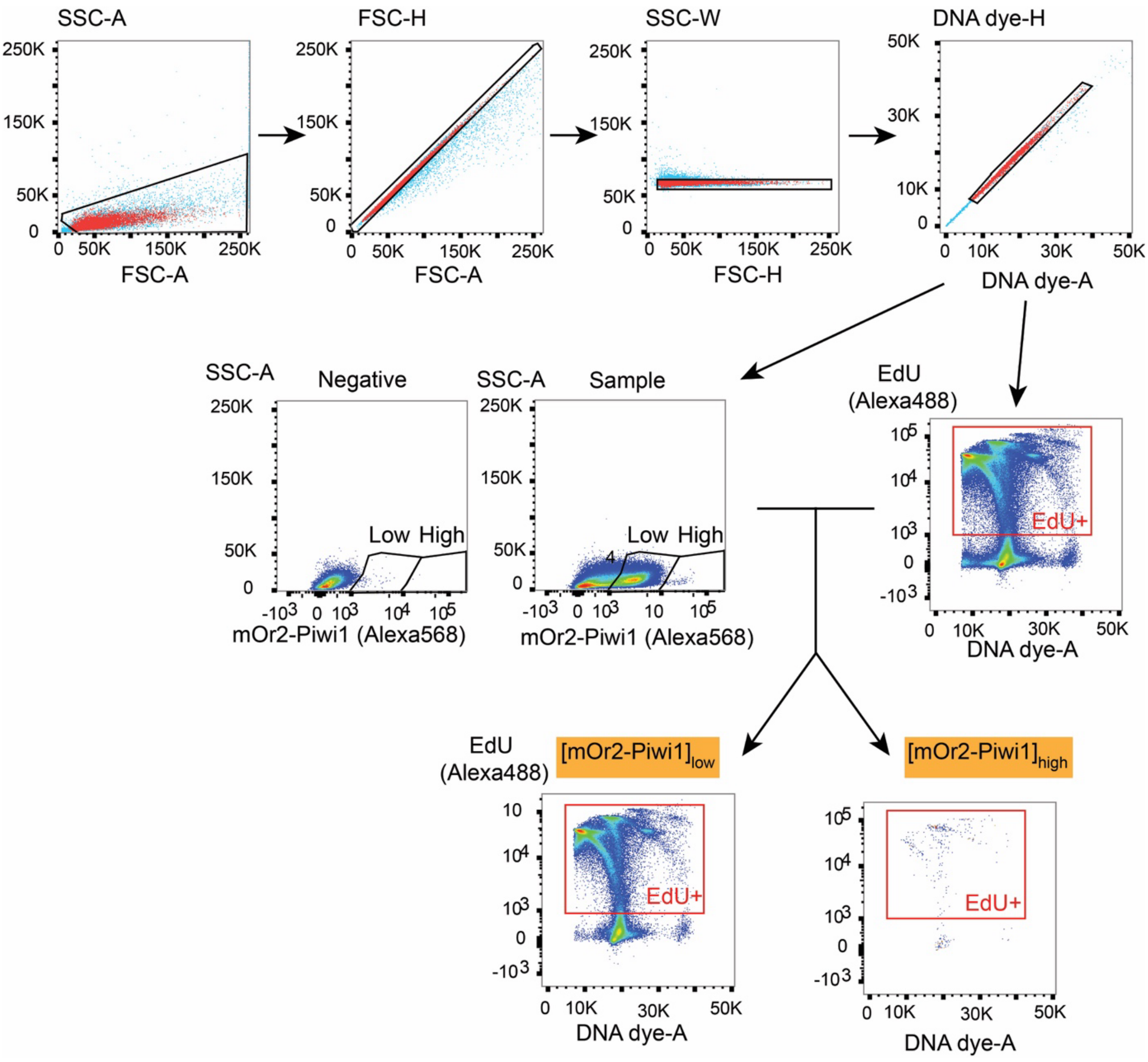

Supplementary Fig. 5. Gating strategy to sort [mOr-Piwi1]$_{high}$ and [mOr-Piwi1]$_{low}$, and their EdU+ subset after 1 day or 3 days-long EdU pulses in juveniles and adults. Using *Nematostella* whole body cell suspensions, we first excluded debris based on size and granularity in the FSC-A/SSC-A gate, with sub-gates based on FSC-A/FSC-H parameters and FSC-A/SSC-W parameters to remove potential cell doublets and high complexity events. Then, we gated events based on DNA dye intensity in area over DNA dye intensity in height on the linear scale. These pre-selected events led to the selection of the pool of cells from which the relative proportion of mOr2-Piwi1 cells and their EdU index were calculated. mOr2-Piwi1 and EdU labeling fluorescence were sub-gated from their respective reference negative controls. In order to sort the EdU positive subfraction, we drew a threshold based on the DNA-A/Alexa488 fluorescence signal of DMSO controls. [mOr2-Piwi1]$_{low}$ were

sorted using a Alexa568/SCC-A fluorescence intensity threshold based on the negative control signal (sample with only secondary antibody staining). The [mOr2-Piwi1]$_{high}$ subpopulation was defined based on the few events presenting high (>10^4) fluorescent intensity (A.U.) and low granularity (SSC-A) using Alexa568/SSC-A parameters. Finally, the EdU index within [mOr2-Piwi1]$_{high}$ and [mOr2-Piwi1]$_{low}$ populations was determined by combining both gates.

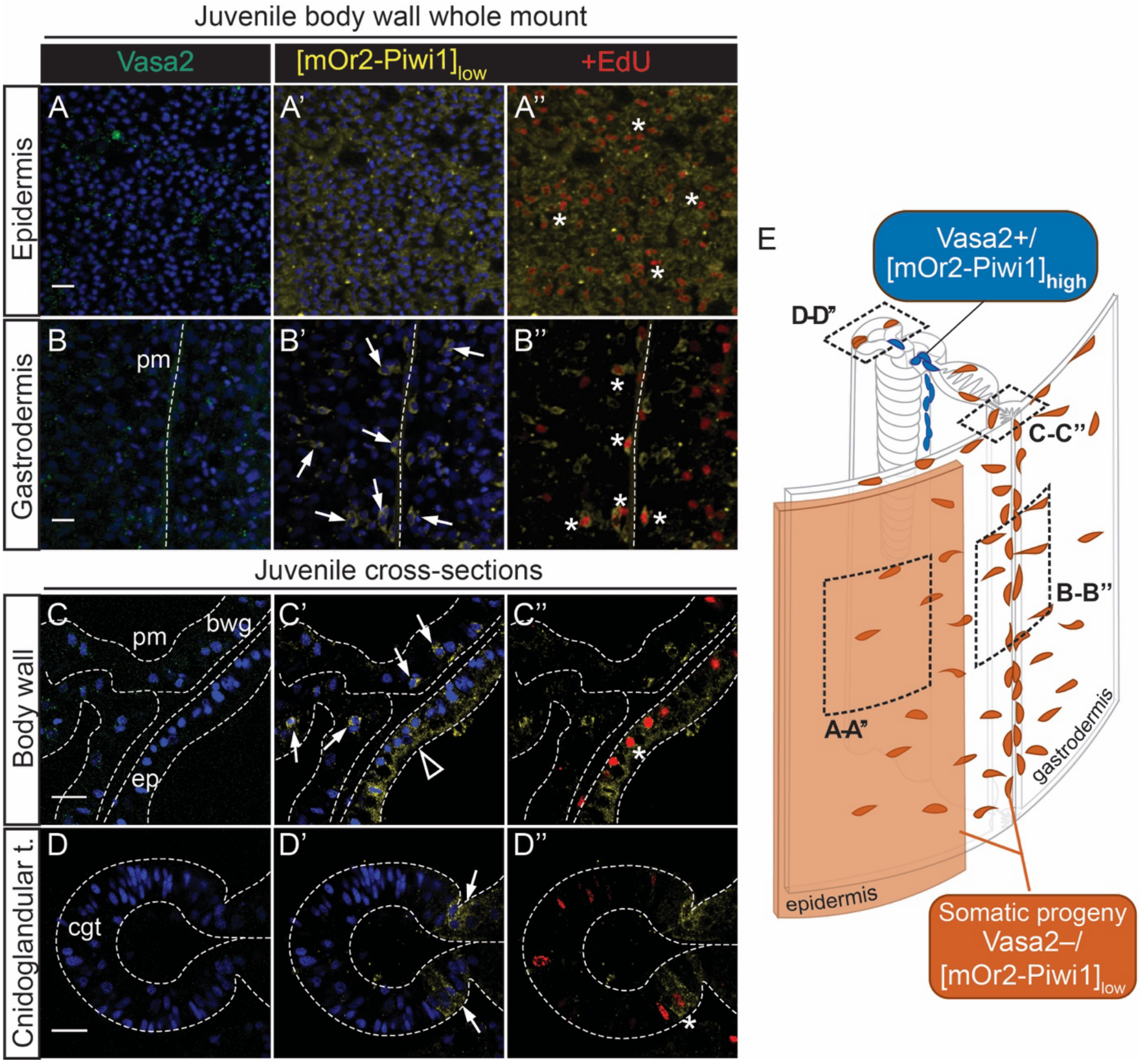

Supplementary Fig. 6. Low levels of mOr2-Piwi1 detected in the epidermis and in abundant proliferative cells of the gastrodermis. (A-D'') Confocal imaging stacks of whole-mount (A-B'') and cross-sectioned (C-D'') *piwi1*^{mOr2} juvenile polyps immunostained for Vasa2 (A-D; green), mOr2-Piwi1 (A'-D''; yellow), and EdU-labelled for S-phase nuclei (A''-D''; 1h EdU pulse, red). No specific Vasa2 protein signal was detected in the epidermis (A), body wall gastrodermis (B, C), parietal muscle (B, C) or cnidoglandular tract (D). However, levels of mOr2-Piwi1 close to detection limit were found ubiquitously in the epidermis (A' and C'; black arrowhead), in gastrodermal, basiepithelial along the parietal muscle and body wall (B', C'; arrows) and at the basis of the cnidoglandular tract (D'; arrows). A larger subset of these [mOr2-Piwi1]$_{low}$ cells were EdU+ (A''-D'', asterisks). **(E)** 3-dimensional schematic of juvenile body column section highlighting the location Vasa2+/[mOr2-Piwi1]$_{high}$ stem/progenitor cells and

their putative Vasa2–/[mOr2-Piwi1]$_{low}$ progeny cells (orange) in *piwi1^{mOr2}* juveniles. Note that *piwi1* mRNA (see Fig. 2D, G) or *mOr2* mRNA (see Supplementary Fig. 7F) were not detected in any of these regions. Blue: Hoechst DNA dye. bwg: body wall gastrodermis; cgt: cnidoglandular tract; ep: epidermis; pm: parietal muscle. Scale bars: 10μm. All experiments performed twice with similar results.

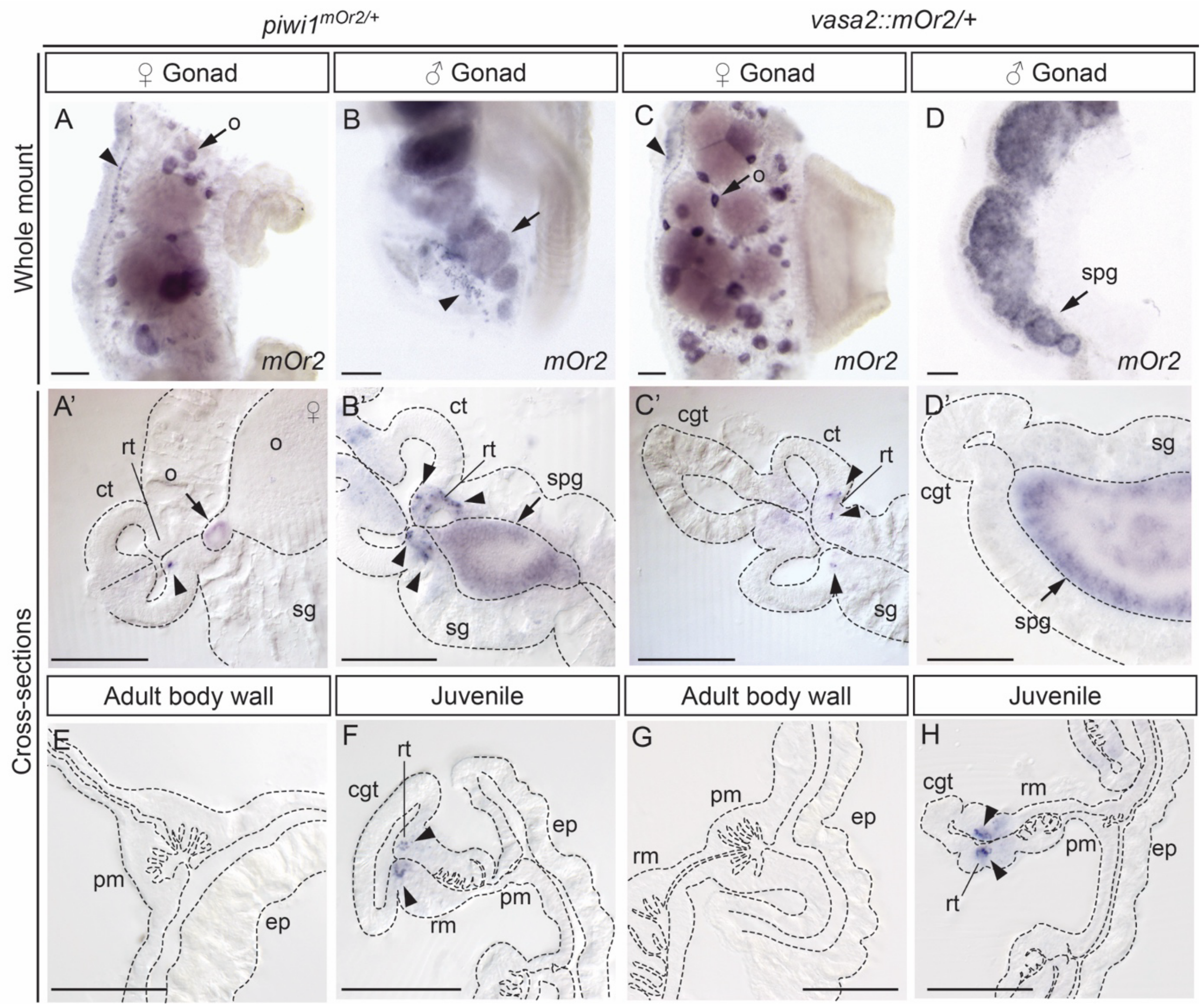

Supplementary Fig. 7. Detection of *mOr2* mRNA in *piwi1*^{mOr2} and *vasa2::mOr2* adult and juvenile mesenteries. (A-H) Whole-mount ISH detecting the *mOr2* gene on dissected tissues (A-D) and cross-sections (A'-H) of *piwi1*$^{mOr2/+}$ (A-B', E, F) or *vasa2::mOr2/*+ (C-D', G, H) of adult (A-D', E, G) or juvenile (F, H) mesenteries. As found for *piwi1* mRNA (see Fig. 1E-F'), *mOr2* is only detected in oocytes (A-A', C; arrows) or spermatogonia (B-B', D-D'; arrows), and in basiepithelial cells along the reticulate tract (A-A', B-B', C-C', arrowheads) in adult polyps of both reporter lines. In juveniles, as found for *piwi1* and *vasa2* mRNA (see Fig. 2D, G, J), *mOr2* signal is restricted to basiepithelial cells located between the basis of the septal filament and the retractor muscle (F, H, arrowheads) in both reporter lines. All scale bars correspond to 100µm. cgt: cnidoglandular tract; ct: ciliated tract; ep: epidermis; o: oocyte; pm: parietal muscle; rm: retractor muscle; rt: reticulate tract; sg: somatic gonad; spg: spermatogonia. Experiments performed twice (adults; A-G) or once (juveniles F, H) with similar results in somatic cells between stages.

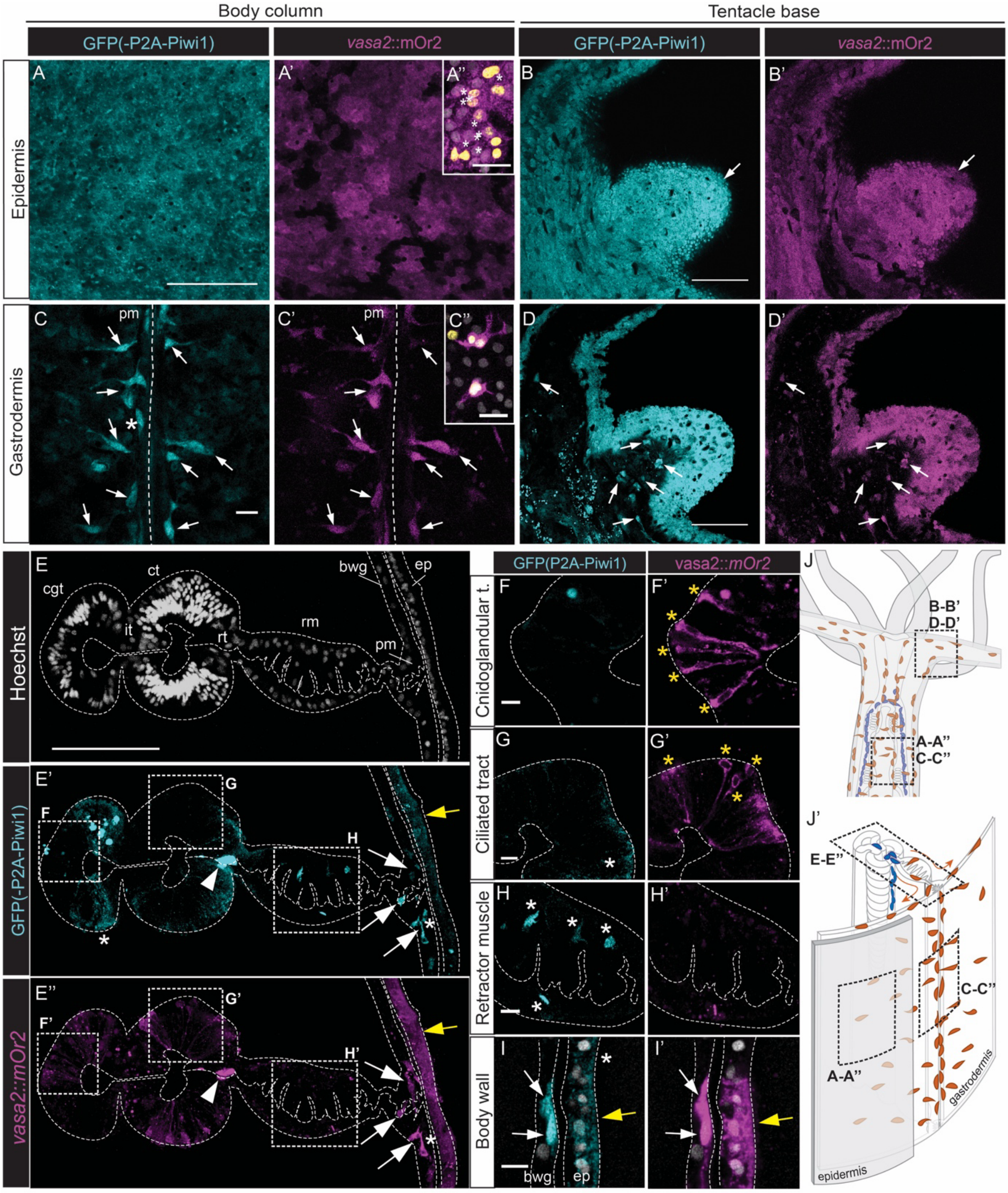

Supplementary Fig. 8. Double *vasa2::mOr2/piwi1$^{P2A\text{-}GFP}$* transgenic reporter line shows broad mOr2 and GFP co-localization, but also subsets of single-labelled, non-overlapping cells. (A-D') *In vivo* confocal imaging stacks of the epidermis (A-B') and gastrodermis (C-D') at the basis of the tentacles and body wall as indicated in (J, J'). As found for Piwi1-mOr2, epidermal GFP appears ubiquitously expressed (A), while different levels of *vasa2::*mOr2 are detectable in patches of cells (A'). Expression of *vasa2*::mOr2 and GFP appear higher in tentacle bud epidermis (B-B'; arrow) than

in adjacent, larger ones. In the gastrodermis, vasa2::mOr2+/GFP+ cells locate along the parietal muscle tracts of the body column (C-C'; arrows) and extend up into the tentacle gastrodermis (D-D'; arrows). (A'', C'') Confocal imaging of a whole mount *vasa2::mOr2* polyp immunostained for mOr2 (magenta) and labelled for EdU (1h pulse, yellow). Note that pairs of cells with similar EdU levels after 1h pulse locate within mOr2+ epidermal cell patches (A''; asterisks) suggesting synchronization of cell cycle phases. Neuron-like mOr2+ cells are found sometimes in duplets in the body wall gastrodermis (C''). **(E-I')** Confocal imaging stacks of cross-sectioned juvenile subpharyngeal mesenteries immunostained for GFP (cyan) and mOr2 (magenta). Boxes in E' and E'' show specific regions presented as close-ups in F-H'. High mOr2 and GFP levels colocalize to stem/progenitor cells in the reticulate tract (E-E''; arrowhead). Lower levels of mOr2 and GFP colocalize to cells within the parietal muscle tract (E'-E''; arrows), body wall gastrodermis (I-I'; white arrows) and epidermis (E-E'', I-I'; yellow arrows). Subsets of GFP+ cells without detectable mOr2 signal (white asterisks) were found in the cnidoglandular and parietal muscle tracts (E'), ciliated tract (G), retractor muscle tract (H) and epidermis (I). Subsets of mOr2+, but GFP– cells (yellow asterisks) located to the cnidoglandular tract (F') and ciliated tract (G'). Grey: Hoechst DNA dye. **(J-J')** Schematic of the oral part of a juvenile polyp (L) and a three-dimensional illustration of a body column section (J') with boxes indicating location of sections and views shown in A-E''. bwg: body wall gastrodermis; ct: ciliated tract; cgt: cnidoglandular tract; ep: epidermis; it: intermediate tract; pm: parietal muscle; rm: retractor muscle; rt: reticulate tract. Scale bars: 50μm (A, B, D, E), 10μm (C) and 5μm (A'', C'', F, G, H, I). Experiments in (A-D') performed three times with similar results. Experiments in E-I' performed once, but results of both fluorophores were consistent with whole-mounts of the same double transgenic line (Fig. 4A-D') or in different transgenic combinations (Fig. 7A,C; Supplementary Fig. S9H', J').

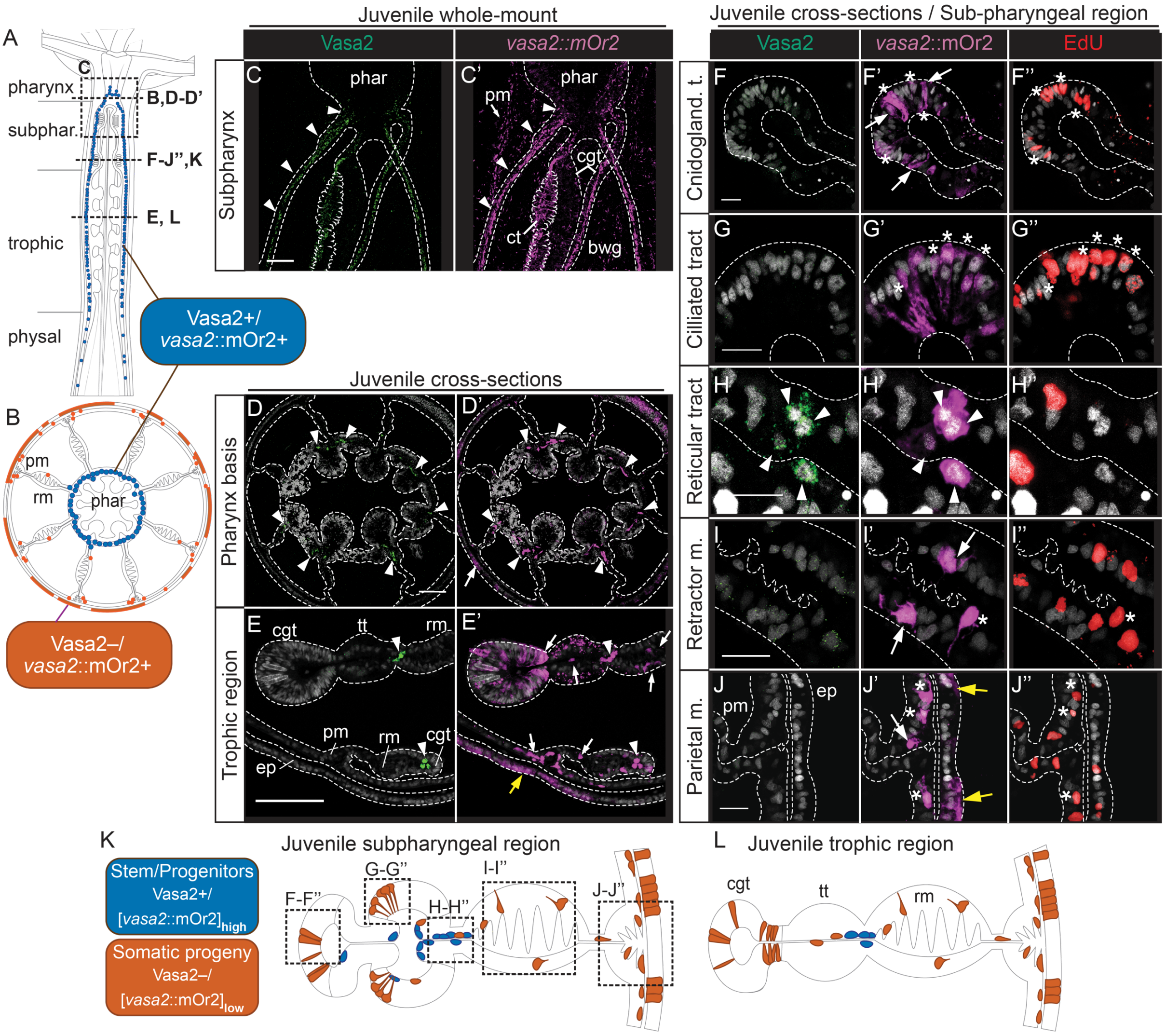

Supplementary Fig. 9. Abundant *vasa2*::mOr2 expression in gastrodermal and epidermal cells that potentially derive from the population of Vasa2+/Piwi1+ stem/progenitor cells in growing juveniles. (A, B, K, L) Summary schematics of location and approximate expression levels of Vasa2 and *vasa2*::mOr2 in longitudinal (A) and cross sections (B, K, L) through pharyngeal (B), subpharyngeal (K) and trophic (L) regions. **(C-J")** Confocal imaging stacks of whole-mount (C-C') or cross-sectioned (D-J") *vasa2::mOr2* juvenile polyps immunostained for Vasa2 protein (C-J, green), mOr2 (C'-J', magenta), and EdU-labelled for S-phase nuclei (F"-J"; 1 day EdU pulse, red). Sections as depicted in (A, B, K, L). Basiepithelial Vasa2+/*vasa2::*mOr2+ stem/progenitor cells (arrowheads) are detected between the septal filament and retractor muscle tract and extend oral-aborally from the basis of the pharynx (A, C-D'), through the subpharyngeal region (A, F-J") to the trophic region (E-E'). Abundant

Vasa2–/mOr2+ cells (C-J'; arrows & asterisks), which are often EdU+ (F''-J''; asterisks), are scattered throughout the gastrodermis. Patches of Vasa2–/mOr2+ cells are also detected in the epidermis (E-E', J-J'; yellow arrows). Grey: Hoechst DNA dye. bwg: body wall gastrodermis; cgt: cnidoglandular tract; ct: ciliated tract; ep: epidermis; it: intermediate tract; m.: muscle; o: oocyte; phar: pharynx; pm: parietal muscle; rm: retractor muscle; rt: reticulate tract; sg: somatic gonad; sp: spermary; spg: spermatogonia; tt: trophic tract. Scale bars: 100μm (C), 50μm (D, E) and 10μm (F-J). Experiments performed twice with similar results.

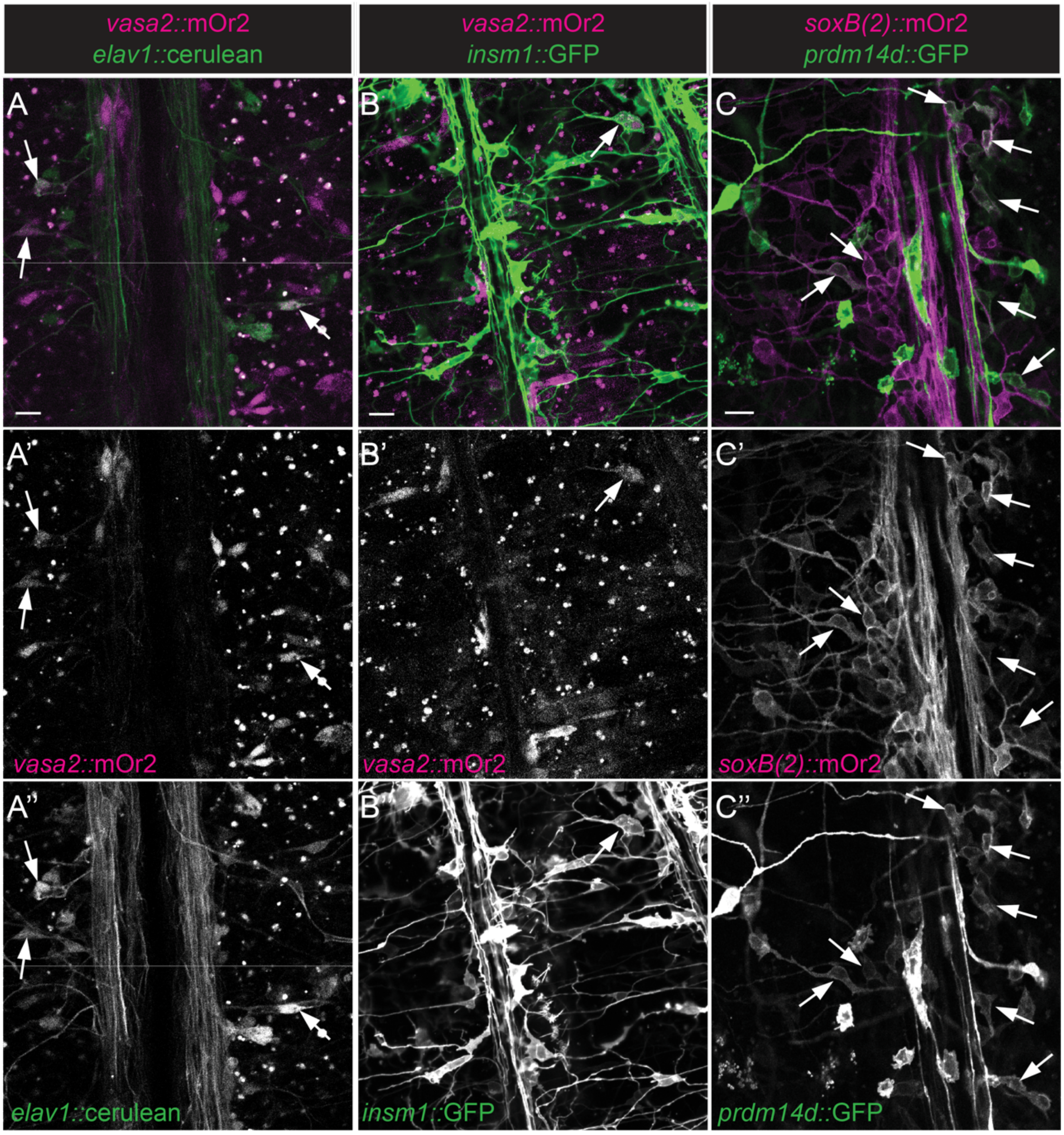

Supplementary Fig. 10. Colocalization between *vasa2::*mOr2 and neuronal reporter lines indicates that a subset of gastrodermal neurons potentially derive from the population of Vasa2+/Piwi1+ stem/progenitor cells in growing juveniles. (A-C'') *In vivo* confocal imaging of juveniles from *vasa2::mOr2/elav1::cerulean* (A-A''), *vasa2::mOr2/insm1::GFP* (B-B'') and *soxB(2)::mOr2/prdm14d::GFP* (C-C'') double reporter lines. In the parietal muscle tract, *vasa2*::mOr2+ cells colocalize with *elav1::*Cerulean+ differentiating neurons (A-A''; arrows) and with *insm1::*GFP+ neuronal or glandular cell precursors (B-B''; arrow). *soxB(2)::*mOr2 and *prdm14d::*GFP colocalize in a subpopulation of neural progenitors along the parietal muscle of a juvenile (C-C'', arrows). Scale bars: 10µm. See text for

further discussion. Experiments performed once but similar and consistent between different individuals, crosses and with published data[4,5,6].

Supplementary References

1. Praher D*, et al.* Characterization of the piRNA pathway during development of the sea anemone *Nematostella vectensis*. *RNA Biol*, 1-15 (2017).

2. Chen C-Y, McKinney SA, Ellington LR, Gibson MC. Hedgehog signaling is required for endomesodermal patterning and germ cell development in the sea anemone *Nematostella vectensis*. *Elife* **9**, e54573 (2020).

3. Steinmetz PR, Aman A, Kraus JE, Technau U. Gut-like ectodermal tissue in a sea anemone challenges germ layer homology. *Nature ecology & evolution* **1**, 1535-1542 (2017).

4. Nakanishi N, Renfer E, Technau U, Rentzsch F. Nervous systems of the sea anemone *Nematostella vectensis* are generated by ectoderm and endoderm and shaped by distinct mechanisms. *Development* **139**, 347-357 (2012).

5. Lemaître QI*, et al.* NvPrdm14d-expressing neural progenitor cells contribute to non-ectodermal neurogenesis in *Nematostella vectensis*. *Nature Communications* **14**, 4854 (2023).

6. Tournière O, Gahan JM, Busengdal H, Bartsch N, Rentzsch F. Insm1-expressing neurons and secretory cells develop from a common pool of progenitors in the sea anemone *Nematostella vectensis*. *Science Advances* **8**, eabi7109 (2022).
