# Supplementary material for: A population of Vasa2 and Piwi1 expressing cells generates germ cells and neurons in a sea anemone
Source: Nat Commun. 2024 Oct 10;15:8765. doi: 10.1038/s41467-024-52806-4 (PMC11464780; doi:10.1038/s41467-024-52806-4)
Supplement: Supplementary file 3 — Reporting Summary [file 41467_2024_52806_MOESM3_ESM.pdf]

nature portfolio

Corresponding author(s): Patrick R. H. Steinmetz

Last updated by author(s): Sep 4, 2024

Reporting Summary

Nature Portfolio wishes to improve the reproducibility of the work that we publish. This form provides structure for consistency and transparency in reporting. For further information on Nature Portfolio policies, see our Editorial Policies and the Editorial Policy Checklist.

Statistics

For all statistical analyses, confirm that the following items are present in the figure legend, table legend, main text, or Methods section.

n/a	Confirmed	
☐	☒	The exact sample size (*n*) for each experimental group/condition, given as a discrete number and unit of measurement
☐	☒	A statement on whether measurements were taken from distinct samples or whether the same sample was measured repeatedly
☒	☐	The statistical test(s) used AND whether they are one- or two-sided<br>*Only common tests should be described solely by name; describe more complex techniques in the Methods section.*
☒	☐	A description of all covariates tested
☒	☐	A description of any assumptions or corrections, such as tests of normality and adjustment for multiple comparisons
☒	☐	A full description of the statistical parameters including central tendency (e.g. means) or other basic estimates (e.g. regression coefficient) AND variation (e.g. standard deviation) or associated estimates of uncertainty (e.g. confidence intervals)
☒	☐	For null hypothesis testing, the test statistic (e.g. *F*, *t*, *r*) with confidence intervals, effect sizes, degrees of freedom and *P* value noted<br>*Give P values as exact values whenever suitable.*
☒	☐	For Bayesian analysis, information on the choice of priors and Markov chain Monte Carlo settings
☒	☐	For hierarchical and complex designs, identification of the appropriate level for tests and full reporting of outcomes
☒	☐	Estimates of effect sizes (e.g. Cohen's *d*, Pearson's *r*), indicating how they were calculated

Our web collection on statistics for biologists contains articles on many of the points above.

Software and code

Policy information about availability of computer code

Data collection	n.a.
Data analysis	FlowJoV10.9 (BD Life Sciences) was used for flow cytometry data analysis.

For manuscripts utilizing custom algorithms or software that are central to the research but not yet described in published literature, software must be made available to editors and reviewers. We strongly encourage code deposition in a community repository (e.g. GitHub). See the Nature Portfolio guidelines for submitting code & software for further information.

Data

Policy information about availability of data

All manuscripts must include a data availability statement. This statement should provide the following information, where applicable:

- Accession codes, unique identifiers, or web links for publicly available datasets
- A description of any restrictions on data availability
- For clinical datasets or third party data, please ensure that the statement adheres to our policy

Source data of flow cytometry data are provided as Supplementary Data 2 with this paper. The flow cytometry files (.fcs) generated for this project are available at the Figshare repository: https://doi.org/10.6084/m9.figshare.26661349.v1 and https://doi.org/10.6084/m9.figshare.26661358.v1. All raw imaging data and transgenic lines available on request.

Research involving human participants, their data, or biological material

Policy information about studies with human participants or human data. See also policy information about sex, gender (identity/presentation), and sexual orientation and race, ethnicity and racism.

Reporting on sex and gender	n.a.
Reporting on race, ethnicity, or other socially relevant groupings	n.a.
Population characteristics	n.a.
Recruitment	n.a.
Ethics oversight	n.a.

Note that full information on the approval of the study protocol must also be provided in the manuscript.

Field-specific reporting

Please select the one below that is the best fit for your research. If you are not sure, read the appropriate sections before making your selection.

☒ Life sciences ☐ Behavioural & social sciences ☐ Ecological, evolutionary & environmental sciences

For a reference copy of the document with all sections, see nature.com/documents/nr-reporting-summary-flat.pdf

Life sciences study design

All studies must disclose on these points even when the disclosure is negative.

Sample size: Sample size was only relevant for flow cytometry, where we used 5-7 biological replicates, which is standard in the field. As our aim was to determine the abundance of certain cell populations, but not between different experimental conditions, no statistical tests were applied and sample size was of now major relevance.

Data exclusions: This was no clinical study, and no data was excluded. Images were selected to show representative examples.

Replication: Flow cytometry experiment were performed on 5-7 biological replicates as described more specifically further below. All other experiments were replicated at least once with the exception of:
• The immunostaining showing that two previously published Vasa2 antibodies completely overlap (Supplementary Figure 2). One of the two antibodies has been used throughout the rest of the manuscript with consistently reproducible results.
• Immunostainings or in vivo detection of vasa2:mOr2 in combination with GFP(P2A-Piwi1) and different neural reporter transgenes in juveniles (Figure 5H-I'; Fig. 7; Supplementary Figure 8E-I' and 10). Due the high number of combinations of different transgenic crosses and antibodies, it was not possible to repeat all different combinations within the remaining contract time of the first author. All results were however consistent with whole-mount in vivo imaging of the same transgenic cross (for Fig. 5H-I'), between different individuals, between different transgenic crosses and with published data of neural reporter lines. Please see the figure legends for more details on 'cross-replication' between lines.
• One in situ hybridisation experiments (vasa2 in juveniles, Fig, 2J) was very challenging and performed only once successfully, but is fully consistent with the location of Vasa2 antibody stainings and Piwi1 expression in Vasa2+/Piwi1+ cells at the same stage.
• Control in situ hybridisation experiments on the mOrange2 gene in piwi1mOr2/+ and vasa2::mOr2/+ was performed twice in adults and only once in juveniles, with the juvenile result confirming the results in adults.

Randomization: There was no allocation to specific experimental groups that were subsequently quantified or compared.

Blinding: We did not test statistical significance between different samples, so blinding was not relevant for any experiments.

Reporting for specific materials, systems and methods

We require information from authors about some types of materials, experimental systems and methods used in many studies. Here, indicate whether each material, system or method listed is relevant to your study. If you are not sure if a list item applies to your research, read the appropriate section before selecting a response.

Materials & experimental systems

n/a	Involved in the study
☐	☒ Antibodies
☒	☐ Eukaryotic cell lines
☒	☐ Palaeontology and archaeology
☐	☒ Animals and other organisms
☒	☐ Clinical data
☒	☐ Dual use research of concern
☒	☐ Plants

Methods

n/a	Involved in the study
☒	☐ ChIP-seq
☐	☒ Flow cytometry
☒	☐ MRI-based neuroimaging

Antibodies

Antibodies used	Both antibodies used were custom-made and gifted by other labs (Technau lab and Gibson lab), as described in the materials and methods. In addition, following commercial antibodies were used: rabbit anti-DsRed 1:100 (Takara Bio Clontech 632496), mouse anti-mCherry 1:100 (Takara Bio Clontech 632543), mouse anti-GFP 1:250 (Abcam Ab1218), rat anti- -Tubulin (YL1/2 clone) 1:100 (Abcam Ab6160) and FluoTag®-X4 anti-GFP (N0304), goat-anti-mouse-Alexa488/568 (LifeTech A11001, A11004), goat-anti-rabbit-DyLight488 (LifeTech 35552), goat-anti-rabbit-Alexa568/647 (LifeTech A11011, A21244) and goat-anti-rat-Alexa633 (LifeTech A21094)
Validation	The custom antibody used nearly in all our experiments was kindly provided by the Technau lab. It is a mono-clonal antibody that was produced by Abmart against short synthetic peptides (AGEDGDRPKP and SAGGGDDDWE) corresponding to Nematostella Vasa2. It was previously validated on Western Blot and by immunofluorescence in doi:10.1080/15476286.2017.1349048. In addition, we validated it by using immunfluorensce and showing that it completely overlaps with the polyclonal Vasa2 antibody published by the Gibson lab (doi:10.7554/eLife.54573).

Animals and other research organisms

Policy information about studies involving animals; ARRIVE guidelines recommended for reporting animal research, and Sex and Gender in Research

Laboratory animals	Nematostella vectensis polyps derive from CH6 females and CH2 males constituting the original culture by Hand & Uhlinger 1992 (doi.org/10.2307/1542110).
Wild animals	The study did not involve wild animals.
Reporting on sex	For all adult data, the sex is indicated. The sex cannot be determined in juveniles, and thus all data shown is from a mix of males and females.
Field-collected samples	The study did not involve sampling from the field.
Ethics oversight	No ethical approval is required for working with using lab-reared the sea anemones Nematostella vectensis for biological research.

Note that full information on the approval of the study protocol must also be provided in the manuscript.

Plants

Seed stocks	N.a.
Novel plant genotypes	N.a.
Authentication	N.a.

Flow Cytometry

Plots

Confirm that:

☒ The axis labels state the marker and fluorochrome used (e.g. CD4-FITC).

☒ The axis scales are clearly visible. Include numbers along axes only for bottom left plot of group (a 'group' is an analysis of identical markers).

☒ All plots are contour plots with outliers or pseudocolor plots.

☒ A numerical value for number of cells or percentage (with statistics) is provided.

Methodology

Sample preparation	Animals were washed with calcium- and magnesium-free Nematostella medium (CMF/NM) followed by CMF/NM containing 0.195% ethylenediaminetetraacetic acid (CMF/NM+E). Animals were then incubated for 5 minutes at 37°C in preheated CMF/NM+E containing 0.25% Trypsin (w/v). Homogenization was performed by pipetting and trypsinization was stopped by adding cold CMF/NM containing 1% BSA and 2.5% of Fetal Bovine Serum. Cells were centrifuged for 5 minutes at 800g and at 4°C, resuspended in 1XPBS/1%BSA, filtered through a pre-wetted 50 µm CellTrics strainer (Sysmex) and fixed with 3.7% formaldehyde for 30 minutes at RT in the dark. Finally, the cell suspension was centrifuged at 800 g for 5 minutes at 4°C and was washed twice with 1XPBS/1% BSA. The final cell pellet was then resuspended in 90% Methanol/10% 1XPBS/1%BSA and stored at -20C.
Instrument	Flow cytometry was performed on a BD LSRFortessa (BD Life Sciences) instrument.
Software	Data was analyzed using FlowJoV10.9 (BD Life Sciences).
Cell population abundance	The fractions of mOr-Piwi1+ and EdU+ cells were analyzed, by sub-gating on Piwi1-mOr and EdU labeling fluorescence with reference to negative controls, respectively.
Gating strategy	Graphical representation of the gating strategy is visualised in Supplementary Figure 5. Briefly, debris was excluded based on size and granularity in the FSC-A/SSC-A gate, cell doublets based on FSC-A/FSC-H parameters, and high complexity events based on and FSC-A/SSC-W parameters. DNA dye intensity in area over height on the linear scale was used to select the pool of cells.

☒ Tick this box to confirm that a figure exemplifying the gating strategy is provided in the Supplementary Information.
